# Meeting patients where they are: improving outcomes in early chronic kidney disease with tailored self-management support (the CKD-SMS study)

**DOI:** 10.1186/s12882-018-1075-2

**Published:** 2018-10-20

**Authors:** Kathryn Havas, Clint Douglas, Ann Bonner

**Affiliations:** 1 School of Nursing, Queensland University of Technology, Victoria Park Rd, Kelvin Grove, Brisbane, QLD 4059 Australia; 2 NHMRC Chronic Kidney Disease Centre for Research Excellence, University of Queensland, St Lucia, Australia; 3 Visiting Research Fellow, Kidney Health Service, Metro North Hospital and Health Service, Brisbane, Australia

**Keywords:** Chronic kidney disease, Self-management, Self-care, Person-centred care, Patient-centred care, Intervention, Patient education

## Abstract

**Background:**

To achieve optimal health outcomes, people with chronic kidney disease must make changes in their everyday lives to self-manage their condition. This can be challenging, and there is a need for self-management support interventions which assist people to become successful self-managers. While interventions have been developed, the literature in this area is sparse and limited by lack of both individualisation and sound theoretical basis. The aim of this study was to implement and evaluate the Chronic Kidney Disease-Self-Management Support intervention: a theory-based, person-centred self-management intervention for people with chronic kidney disease stages 1–4.

**Methods:**

A single-sample, pre-post study of an individualised, 12-week intervention based upon principles of social-cognitive theory and person-centred care was conducted with patients attending outpatient renal clinics in Queensland, Australia (*N* = 66). Data were collected at T0 (pre-intervention) and T1 (post-intervention). Primary outcomes were self-efficacy and self-management behaviour.

**Results:**

There were significant, small-to-medium improvements in primary outcomes (self-efficacy: mean difference + 0.8, 95% CI 0.3–1.2, *d* = 0.4; self-management behaviour: mean difference + 6.2, 95% CI 4.5–7.9, *d* = 0.8). There were further significant improvements in secondary outcomes (blood pressure, disease-specific knowledge, physical activity, fruit and vegetable consumption, alcohol consumption, health-related quality of life, psychological distress, and communication with healthcare providers), with effect sizes ranging from negligible to large (all *p*s < .05).

**Conclusions:**

Social-cognitive theory shows promise as a framework for providing effective person-centred self-management support to patients within this population, and longer-term evaluation is needed.

**Trial registration:**

Australian New Zealand Clinical Trials Registry ACTRN12618000066280. Retrospectively registered 17/01/2018.

**Electronic supplementary material:**

The online version of this article (10.1186/s12882-018-1075-2) contains supplementary material, which is available to authorized users.

## Background

Chronic kidney disease (CKD) is a significant burden to those with the disease [1] and healthcare systems [2]. Prevalence is rising, due to increasing rates of diabetes, obesity, and hyptertension [3], and this is predicted to continue [4], with variation across different socioeconomic groups [5]. Burden is particularly high in end-stage kidney disease (ESKD), which requires expensive, time-consuming kidney replacement therapies (KRT; dialysis or transplantation). In earlier stages of CKD, there is an opportunity for interventions to slow progression and improve outcomes. Effective self-management (in areas including diet, physical activity, medication, and smoking and alcohol reduction) impacts health outcomes [6, 7]; therefore, interventions which improve adherence in these areas have potential to improve outcomes for people with CKD. However, self-management support (SMS) intervention studies for people with CKD, especially earlier stages, are rare and limited by methodological and reporting issues [8–10]. Furthermore, difficulties have been identified in recruiting [10, 11] and sustaining [12, 13] participation. The current study builds upon the extant literature to address some of these problems, drawing on patient preferences for SMS and a theoretical framework for behavioural change. The personalised intervention reported here (the Chronic Kidney Disease Self-Management Support program – CKD-SMS) aimed to meet patients where they are, developing goals and tailoring support to improve knowledge, skills, and confidence in self-managing CKD in ways that are congruent with their current knowledge, skills, and engagement with regard to managing their health.

**Fig. 1:**
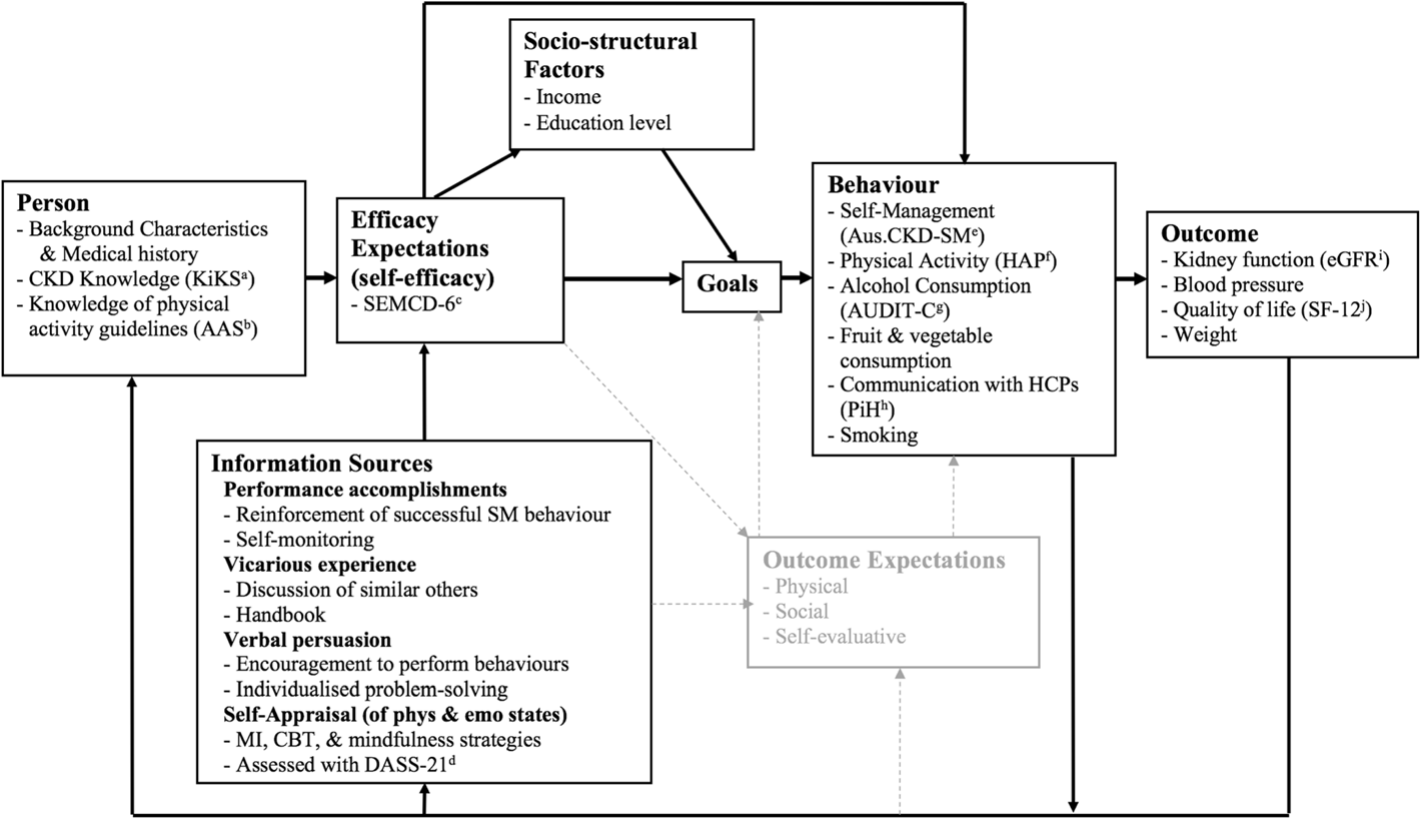
SCT model (adapted from [19]. ^a^Kidney Knowledge survey [31]; ^b^Four items from the Active Australia Survey [33]; ^c^Self-Efficacy for Managing Chronic Disease 6-Item Scale [29]; ^d^Depression, Anxiety, and Stress Scales 21-Item Version [32]; ^e^CKD Self-Management Instrument Australian version [30]; ^f^Human Activity Profile [35]; ^g^Alcohol Use Disorders Identification Test Consumption Questions Scale [37]; ^h^Two items from Partners in Health Scale [36]; ^i^SF-12v2, Australian version [34]

Despite the importance of theory-driven intervention [14], efforts to improve CKD self-management have been largely atheoretical [8]. Additionally, not all behaviour change theories are suited to guide SMS. Some fail to account for the role of emotional states, assuming instead that all behaviour has purely a rational basis [15, 16]. One effective way of improving CKD self-management is by improving self-efficacy (confidence in ability [17]) to manage the disease [18, 19]. Social-cognitive theory (SCT [17]) provides a framework for mechanisms of change in a self-efficacy-based model of behaviour. Within a SCT framework (see Fig. 1), self-efficacy is at the heart of behaviour change, and amount of change in behavioural, psychological, and clinical outcomes depends on factors including baseline knowledge and skills, past experiences self-managing, and available coping resources.

Attempts have been made to deliver SCT-based self-management interventions within the CKD literature. However, these have been underdeveloped in attempts to manipulate self-efficacy, tending to focus on only one mechanism of self-efficacy change (including studies focused solely on verbal persuasion, the weakest mechanism [17]), or have not described theoretical reasons for change at all [10]. Furthermore, these studies (like the broader CKD literature) are limited by design and reporting problems [8–10]. A major gap is lack of individualisation, as the CKD population are heterogeneous in terms of support needs and capacity to participate in interventions [20, 21]. Additionally, patient activation (engagement with self-management and treatment [22]) in chronic illness is developmental, passing through several stages (from understanding that participation is important, to ability to maintain effective self-management under stress).

In contrast to a “one-size-fits-all” approach, a person-centred approach [23] to SMS requires an understanding of support desires of the target population and a willingness to take into account individual circumstances, capacity, activation, and motivation while working collaboratively with patients to generate and work towards personally meaningful and attainable goals. People at different stages of activation are likely to benefit from different types of SMS to improve self-efficacy, ranging from basic education to complex problem-solving and skill-development [22]. This exploratory study aimed to evaluate a person-centred, theory-based intervention to improve self-management in people with stage 1–4 CKD (the CKD-SMS). It was hypothesised that the CKD-SMS would lead to increased self-efficacy, CKD knowledge, and engagement in desirable behaviours (CKD self-management, physical activity, fruit and vegetable consumption, and effective communication with healthcare providers (HCPs)), as well as reduction in emotional distress and undesirable behaviours (smoking and alcohol consumption). According to SCT, this should lead to improvements in CKD outcomes (blood pressure (BP), health-related quality of life (HRQoL), weight, and estimated glomerular filtration rate (eGFR)).

## Methods

### Design and participants

This study was a prospective, single-sample, pre-post design. This design was selected over a randomised-controlled trial (RCT) due to aforementioned known challenges with recruitment and participant retention amongst this population, which are such that recruiting and retaining a sample to adequately power a 2 × 2 research design was not feasible. Furthermore, the flexibility that is inherent in a person-centred intervention required flexible appointment and rescheduling options, which are incompatible with a highly controlled RCT design. One-sample, repeated-measures designs are common in this field due to the above reasons (e.g., [24–27]), while those studies which have chosen an RCT design cite their small sample sizes as a weakness [28, 29]. Pre-post designs make valuable contributions to the literature when experimental designs are not feasible due to practical constraints such as these [30]. The Transparent Reporting of Evaluations with Nonrandomized Designs (TREND) statement was used to guide reporting [31], and the Template for Intervention Description and Replication (TIDieR) [32] was used to ensure adequate intervention description. Fifty-six participants were required to have 95% power to detect a medium effect (*d* = 0.50 [33]) in a paired t-test, assuming a 5% significance level (two-tailed; calculated using G*Power [34]). These estimates were based upon results of Su and colleagues [19] who used the same primary outcome (the Self-Efficacy for Managing Chronic Disease 6-Item Scale; SEMCD-6) with a similar population to evaluate a SCT-based intervention. Allowing for approximately 30% attrition, we recruited 78 participants.

**Fig. 2:**
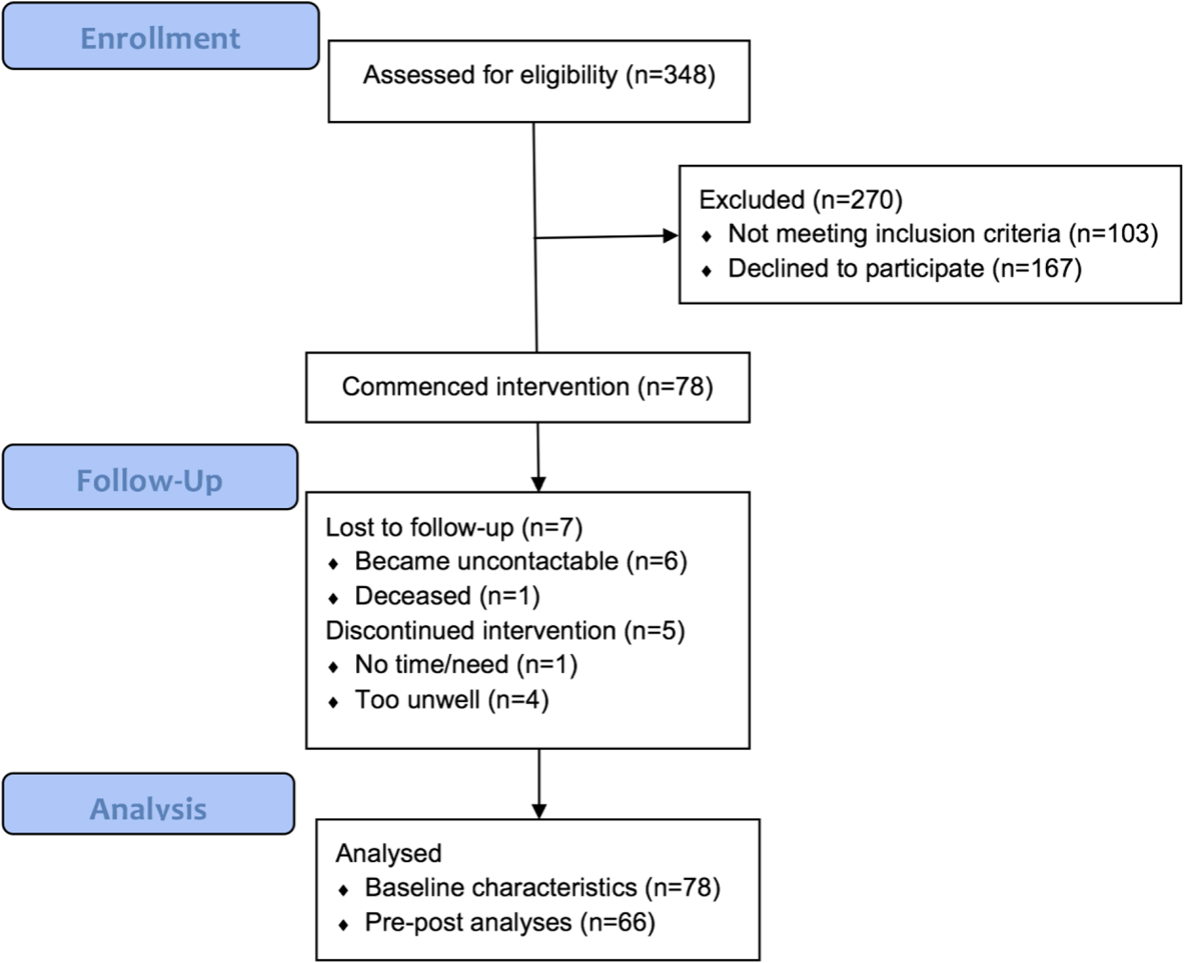
Participant flow

Patients who attended five nephrologists’ clinics across two public sector outpatient renal clinics in Queensland, Australia were screened by clinic staff for eligibility. These sites are general nephrology outpatient clinics, where patients are typically referred by their General Practitioner or family physician when their eGFR decreases below 60 mL/min/1.73m^2^ or sometimes earlier in the case of sudden kidney function deterioration. Inclusion criteria were: diagnosis of CKD; eGFR ≥25 mL/min/1.73m^2^ (so participants would not have commenced pre-dialysis education); ≥18 years of age; and ability to understand English. Exclusion criteria were: cognitive impairment which would inhibit participation (as determined by clinic staff); inability to be followed up (> 60 km from researcher’s location); and already receiving extensive CKD SMS through select Queensland clinics known to provide enhanced SMS as part of their renal care. Participant recruitment and participation can be seen in Fig. 2. Data were collected at T0 (baseline) and T1 (13 weeks; 1 week after intervention completion). Due to financial constraints, blinded data collection was not possible.

### Procedure

Recruitment took place between April and December 2016, with follow-up completion in March 2017. Flyers were sent with appointment letters, and verbal consent was obtained for the researcher to approach when patients attended appointments. A systematic recruitment protocol was implemented whereby all eligible patients were approached consecutively by a male or female researcher and provided study information prior to consenting. Participants commenced study instruments, which took approximately 20–30 min to complete, and finalised these at first intervention appointment. The principal investigator, a researcher in CKD self-management with a background in psychology, delivered intervention sessions over a 12-week period. One week after final intervention session, participants completed study questionnaires again face-to-face with the researcher.

### Intervention: The CKD-SMS

The intervention was highly individualised, based upon principles of person-centred care (PCC) and SCT. Expert input was sought (from nephrologists, renal nurses, academic specialists in CKD, and people with CKD) regarding intervention and resource design. The design of the CKD-SMS was also informed by our previous work investigating SMS desires of people with CKD [20, 21], conducted with the explicit goal of informing intervention development. An outline of the purposes and procedures of CKD-SMS sessions can be seen in Additional file 1.

Participants engaged in two face-to-face intervention sessions with the principal researcher, at week 1 and week 12, which took place in a mutually convenient location (most often the participant’s home or workplace, 80.8%). Length of these sessions ranged from 20 to 90 min (*M* = 44 min), dependent upon individual need. Between face-to-face sessions, participants nominated preferred frequency of phone sessions (weekly, fortnightly, or monthly), with most (71.2%) opting for monthly sessions. Phone sessions ranged from five to 60 min, but were mostly brief (*M* = 12 min). At the first session, participants and researcher collaborated to generate individualised goals in areas including: diet; physical activity; communication with HCPs and engagement with treatment; emotional distress; maintaining roles; and understanding CKD and laboratory results. During the course of the intervention, techniques from SCT (performance accomplishment reinforcement, vicarious experience, and verbal persuasion) were used to assist participants achieve their goals, as were motivational interviewing, cognitive-behavioural, and mindfulness techniques as appropriate (see Additional file 2 for examples of how these strategies were used).

Participants were given a handbook (adapted with permission from Kidney Health Australia’s “Living with Reduced Kidney Function” handbook [35]) to accompany the intervention, which was used to prompt discussion. Participants also received self-monitoring and note-taking handouts,^1^ and were encouraged to request further resources. Participants were encouraged to invite family members or friends to attend sessions if desired. One week after the final face-to-face intervention session, participants completed study questionnaires again face-to-face with the researcher.

### Intervention Fidelity

All included participants engaged in at least two telephone sessions and two face-to-face sessions during the intervention period. However, during the program, there were 23 instances of participant failure to attend a scheduled face-to-face appointment and 15 instances in which participants were unable to be contacted for scheduled telephone sessions (despite multiple attempts). The person-centred nature of the program meant that flexibility was possible and most participants were able to be followed up and to continue participation in the program.

### Outcomes and measurement

We hypothesised that the CKD-SMS would improve behavioural and patient outcomes, with associated increased knowledge and self-efficacy and reduced emotional distress. Study measures are detailed in Table 1. Primary outcomes were self-efficacy and self-management, assessed using the SEMCD-6 [36] and the Australian version of the Chronic Kidney Disease Self-Management Instrument (Aus.CKD-SM) [37], respectively. Secondary outcomes were: HRQoL; CKD knowledge; emotional distress; understanding of physical activity guidelines and engagement in physical activity; fruit and vegetable consumption; communication with HCPs; alcohol use; and physiological measures (BP, weight, and eGFR). Additional instruments were: demographic and clinical record form (T0 only); the Kidney Knowledge Survey (KiKS) [38]; the Depression, Anxiety, and Stress Scales 21-Item Version (DASS-21) [39]; four items from the Active Australia Survey (AAS) [40] assessing understanding of physical activity guidelines; the SF-12v2 (Australian version) [41]; the Human Activity Profile (HAP) [42]; two items from the Partners in Health Scale (PiH) [43]; two questions assessing fruit and vegetable consumption; and the Alcohol Use Disorders Test Consumption Questions Scale (AUDIT-C) [44]. Available clinical information at T0 and T1 was gathered from electronic medical records. The Charlson Comorbidity Index (CCI) [45] was used to calculate comorbidity score.

**Table 1:** Study instruments

Outcome	Instrument	Reliability/Validity	Items and Scoring
Background and Clinical Details	Background and clinical details	N/A	10 items assessing background characteristics; 13 items assessing clinical characteristics.
Comorbidity	Charlson Comorbidity Index (CCI) [45]	Valid in predicting risk of death from comorbid disease [45].	Combines age and number of pre-determined, serious comorbidities into a single index. All participants in this study started with a minimum score of 2 for CKD.
CKD Knowledge	Kidney Knowledge Survey (KiKS) [38]	Adequate internal consistency (Kuder-Richardson-20 = .7) [38]; investigates knowledge of important CKD topics such as function, treatment, and BP targets. α = .7 and .8 in current study.	28 multiple-choice items; Maximum score = 28.
Understanding of Physical Activity Guidelines	Four items from Active Australia Survey (AAS) [40]	Assesses understanding of/agreement with physical activity guidelines. Overall instrument has good-excellent reliability (Spearman’s Rho 0.5–0.8) [44], and items used showed high reliability in this study (α = .9).	4 Likert Scale items (1 = *Strongly Disagree* – 5 = *Strongly Agree*); Maximum score = 20.
Self-Efficacy to Manage Chronic Disease	Self-Efficacy for Managing Chronic Disease (SEMCD-6) [36]	High internal consistency (α = .9), scales upon which instrument is based sensitive to change in disease-related self-efficacy in response to intervention [36]. α = .9 and .9 in current study.	6 items scored 1–10. Overall scale score = mean of items 1–10.
Depression, Anxiety, and Stress	Depression Anxiety Stress Scales (DASS-21) [39]	Distinguishes well between psychological disorders and has good-excellent internal consistency (α = .9) [73]. α = .8 - > .9 in current study.	21 Likert Scale items (0 = *Never* – 3 = *Almost Always)*. Three subscales and overall scale. Scores reported are for 21-item version and must be doubled to be compared to 42-item version scores or cut-offs.
CKD Self-Management Behaviour	Chronic Kidney Disease Self-Management Instrument – Australian Version (Aus.CKD-SM) [37]	Adequate internal consistency (α = .7–.85) [37]. α = .6–.9 in current study.	17 Likert Scale items (1 = *Never* – 4 = *Always*). Four subscales and overall scale.
Physical Activity	Human Activity Profile (HAP) [42]	Used extensively in chronic disease research [74]; adequate test-retest reliability (α = .8), content validity [42]. α = .8-. > 9 in current study.	94 items rated “*still doing*”, “*stopped doing*” and “*never did*”. Maximum activity score, adjusted activity score, and four research subscales.
Alcohol Use	Alcohol Use Disorders Identification Test-Consumption Questions (AUDIT-C) [44]	Effective in detection of heavy drinking and alcohol abuse or dependence [44]. α = .7 in current study.	Three multiple-choice questions with five response options scored 0–4; Maximum score 12; clinical cut-off of 3 for women and 4 for men.
Fruit and Vegetable Consumption	Two questions about fruit and vegetable consumption yesterday	Directly assesses consumption of fruit and vegetable on previous day.	Two questions assessing serves of fruit and vegetables eaten on previous day, added together for total.
Communication with Healthcare Providers	Two items from the Partners in Health Scale (PiH) [43]	Overall scale has demonstrated construct and face validity and internal consistency (α = .9) and inter-rater reliability [43]. α = .6 and .8 in current study.	Two Likert Scale items (0 = *Never* – 8 = *Always*). Maximum score = 16.
Health-Related Quality of Life	Short Form Health Survey-12-Item Australian v2; (SF-12) [75]	Conceptually and empirically validated [76]; adequate test-retest reliability (α = .8–.9).	12 Likert Scale items (scales vary from 3- to 5-point). Eight subscales contribute to two overall composite scores.

### Data analysis

Data were analysed using IBM SPSS Statistics version 23 [46]. Descriptive statistics were generated for background and clinical data, and T0 and T1 results on patient-reported instruments and clinical measures were compared. Descriptive statistics are presented as frequencies and/or range, mean (*M*) or median (*Mdn*), and standard deviation (SD) or interquartile range (IQR), as appropriate. Where data met assumptions, paired-samples *t*-tests were conducted to assess change. Where data did not meet assumptions, non-parametric equivalent tests (Wilcoxon Signed-Rank tests) were conducted. Between-groups *t*-tests and Fisher’s exact tests were performed to assess for baseline differences between those who completed the intervention and those who did not. Mean differences (Diff) and 95% confidence intervals (CI), along with effect sizes (*d* or *r*) and statistical significance (at *p* < .05) are reported for all pre-post-intervention analyses.

## Results

### Sample characteristics and participant flow

Background and clinical characteristics of the original sample (*N* = 78) can be seen in Tables 2 and 3. Slightly over half the sample were female, and age ranged from 25 to 84, with a mean of 57.6. Most participants were born in Australia and predominantly spoke English at home, and two participants identified as Aboriginal or Torres Strait Islander (ATSI). Approximately half (47.4%) of participants had a high-school or lower level of education and the same number were currently employed. Most had CKD stage 2 or 3, and a third had been living with CKD for 10 years or longer, while some had been diagnosed as recently as 4 months ago.

**Table 2:** Background characteristics

Variable	Frequency (%)
Gender	
Male	31 (39.7)
Female	47 (60.3)
Age Range: 25–84 *M =* 57.6 SD = 16.7	
25–39	14 (17.9)
40–59	25 (32.1)
60–79	33 (42.3)
≥ 80	6 (7.7)
Place of Birth	
Australia	54 (69.2)
New Zealand	4 (5.1)
South-east Asia	4 (5.1)
Europe	6 (7.7)
Other	10 (12.8)
Main Language	
English	73 (93.6)
Other	5 (6.4)
ATSI^a^ Status	
Identifies as Aboriginal	2 (2.6)
Identifies as neither ATSI	76 (97.4)
Marital Status	
Single	15 (19.2)
Married/Defacto	50 (64.1)
Divorced	11 (14.1)
Widowed	2 (2.6)
Years of Education Range: 0^b^ – 24 *M =* 12.9 SD = 3.9	
Highest Educational Qualification Attained	
Less than Grade 10 Equivalent	8 (10.4)
Grade 10 or Equivalent	22 (28.6)
Grade 12 or Equivalent	7 (9.1)
TAFE Qualification/Certificate/Diploma	21 (27.3)
Undergraduate Degree (Bachelors)	14 (18.2)
Masters Degree	3 (3.9)
Doctoral Degree (Including PhD)	2 (2.6)
Annual Household Income	
< $20,000	10 (12.8)
$20,000 - $39,999	23 (29.5)
$40,000 - $59,999	8 (10.3)
$60,000 - $79,999	7 (9.0)
$80,000 - $99,999	8 (10.3)
$100,000 - $119,999	9 (11.5)
$120,000+	6 (7.7)
Don’t Kno*w*/*W*ould Rather not say	7 (9.0)
Employment Status	
Unemployed	9 (11.5)
Casual	2 (2.6)
Part Time	8 (10.3)
Full Time	25 (32.1)
Retired	32 (41.0)
Other (Employed)	2 (2.6)

^a^*ATSI* Aboriginal or Torres Strait Islander

^b^One participant reported receiving no formal education during her youth in Southeast Asia

**Table 3:** Clinical characteristics

Variable	Frequency (%)
CKD Stage	
1	12 (15.6)
2	22 (28.6)
3A	15 (19.5)
3B	20 (26.0)
4	8 (10.4)
eGFR^a^ Range: 25- > 90 *M* = 57.5 SD = 22.3	
Creatinine μmol/L Range: 48–259 *M* = 116.3 SD = 45.4	
Time Since Diagnosis (Self-Reported) Range: 4 months – 33 years *Mdn* = 5 years QR = 26.3–120.0 (months)	
≤ 12 months	11 (14.5)
12 years 1 month - 3 years	13 (17.1)
3 years, 1 month - 5 years	15 (19.7)
5 years, 1 month < 10 years	12 (15.8)
≥ 10 years	25 (32.9)
Unknown	2 (2.6)
Cause of CKD	
Renovascular	13 (16.9)
Glomerulonephritis	13 (16.9)
Diabetes Mellitus (I or II)	12 (15.6)
Systemic Lupus Erythematosus	8 (10.4)
Other	25 (32.5)
Unknown	6 (7.8)
Charlson Comorbidity Index Score Range: 2–11 *Mdn* = 5.5 IQR = 3.3–8.0	
2–5	36 (50.0)
6–9	31 (43.1)
10+	5 (6.9)
Smoking Status	
Non-smoker	43 (55.1)
Ex-smoker	28 (35.9)
Current Smoker	7 (9.0)
Current Medications Range: 1–14 *Mdn:* 6	

^a^CKD-EPI Creatinine Equation [77]

At baseline, the only difference across background and clinical characteristics and scores on questionnaires between those who completed the intervention and those who did not was that non-completers reported more effective communication with HCPs (*p* = .04).

### Intervention outcomes

All participants displayed improvement in one or more outcomes between T0 and T1. Details of overall group pre-post intervention changes can be seen in Table 4. Participants identified one or more goals, the most common being overall self-management support (identified 30 times) and knowledge (identified 22 times). The highest percentage of improvement in identified areas was attained for those who set goals of improving knowledge (86.4%) or overall self-management (80.0%).

**Table 4:** Pre-post changes in primary and secondary outcomes

	Time – Mean (SD)				
	Baseline	12 weeks	Diff (95% CI)	*d*	*p*
Person Variables					
KiKS	17.0 (5.0)	20.7 (3.8)	3.7 (2.7–4.8)	0.8	<.001
Understanding of Physical Activity Guidelines	16.4 (2.5)	17.2 (2.3)	0.9 (0.1–1.6)	0.4	.02
Self-Efficacy					
SEMCD-6	6.7 (2.1)	7.44 (1.9)	0.8 (0.3–1.2)	0.4	<.01
Emotional Distress/Self-Appraisal					
DASS-21					
*Depression*^ab^	4.2^c^ (4.1^d^)	3.0^c^ (3.3^d^)	1.2 (0.1–2.1)	0.3^e^	.03
*Anxiety*^ab^	4.6^c^ (4.1^d^)	4.4^c^ (4.1^d^)	0.2 (−0.91–1.3)	0.1^e^	.70
*Stress*^ab^	5.7^c^ (4.2^d^)	4.5^c^ (3.6^d^)	1.2 (0.3–2.1)	0.3^e^	.01
Behaviour					
Aus.CKD-SM	47.0 (8.7)	53.2 (7.5)	6.2 (4.5–7.9)	0.8	<.001
*Self-Integration*	13.7 (3.3)	15.8 (2.9)	2.2 (1.5–2.9)	0.7	<.001
*Seeking Support*	8.3 (2.7)	9.2 (2.7)	1.0 (0.4–1.5)	0.4	<.001
*Adherence to Lifestyle Modifications*	12.4 (2.8)	13.7 (2.7)	1.4 (0.8–2.0)	0.5	<.001
*Problem-Solving*	12.7 (2.7)	14.4 (2.1)	1.7 (1.1–2.3)	0.7	<.001
HAP					
*Maximum Activity Score*	69.2 (15.6)	72.0 (14.8)	2.8 (0.6–5.0)	0.2	.01
*Adjusted Activity Score*	59.4 (21.5)	62.4 (19.3)	3.0 (0.6–5.3)	0.2	.02
*Self-care*	7.5 (1.6)	7.5 (1.5)	< 0.1 (−0.2–0.2)	< 0.1	.89
*Personal/Household Work*	18.7 (6.6)	19.4 (5.9)	0.7 (− < 0.1–1.5)	0.1	.06
*Entertainment/Social*	9.5 (2.8)	10.0 (2.6)	0.5 (< 0.1–0.9)	0.2	.04
*Independent Exercise*	8.0 (6.5)	9.2 (6.8)	1.2 (0.1–2.2)	0.2	.04
AUDIT-C	2.4 (2.7)	2.0 (2.3)	0.3 (0.1–0.6)	0.1	.01
Fruit and Vegetables Consumed Yesterday^a^	2.3 (1.5)	6.3 (3.0)	4.0 (3.3–4.8)	1.7	<.001
Communication with HCPs^f^	13.4 (2.9)	14.3 (2.3)	0.7 (0.2–1.6)	0.3	.01
Cigarettes per day (*n* = 4)	16.3 (4.8)	16.3 (4.8)	0.0 (−6.5–6.5)	< 0.001	.99
Outcomes					
eGFR (*n* = 46)	55.2 (24.1)	51.3 (24.2)	3.9 (1.0–6.9)	0.7	.01
BP (*n* = 39) 88.5% at target (T0 and T1)					
*Systolic*	129.6 (23.3)	120.7 (15.8)	8.9 (2.9–14.9)	0.7	<.01
*Diastolic*	74.8 (11.1)	70.5 (9.6)	4.3 (0.9–7.8)	0.6	.01
SF12					
*PCS*	41.3 (11.1)	44.5 (8.7)	3.2 (0.9–5.4)	0.3	.01
*MCS*	51.9 (9.8)	50.9 (9.6)	−1.1 (−3.8–1.6)	0.1	.43
Weight (*n* = 37)	87.4 (30.6)	87.8 (30.6)	−0.4 (−1.1–0.4)	0.3	.32

*SD* standard deviation, *Diff* mean difference, *CI* confidence interval

*d* = Effect size (small ≥0.2; medium ≥0.5; large ≥0.8 [40])

^a^*n* = 56

^b^Wilcoxon Signed-Rank test results reported to deal with effects of violations of t-test assumptions

^c^Median

^d^IQR

^e^*r* = Effect size (small ≥0.1; medium ≥0.3; large ≥0.5 [40])

^f^*n* = 65

### Self-efficacy and self-management behaviour

Comparison of T0 and T1 SEMCD-6 mean scores revealed that participants’ self-efficacy to manage CKD improved significantly (Diff = 0.8, CI = 0.3–1.2, *p =* .001), though the effect size was small (*d* = 0.4). Scores on the Aus.CKD-SM indicated that engagement in CKD self-management behaviours also increased during the study period, with significant improvement in scores on the overall instrument (Diff = 6.2, CI = 4.5–7.9, *p* < .001) and all individual subscales (self-integration; seeking support; adherence to lifestyle modifications; and problem-solving; all *p*s < .001), with effects ranging from small (for seeking support; *d* = 0.4) to medium (for all other subscales and overall instrument, *d*s ranging from 0.5 to 0.8). Almost all participants (63; 95.5%) displayed improvement in one or both primary outcomes.

### CKD knowledge

Change in mean scores on the KiKS revealed significant improvement in CKD knowledge over the course of the intervention (*p* < .001), with a large effect size (*d* = 0.8).

### Knowledge of physical activity guidelines and engagement in physical activity

Understanding of physical activity guidelines increased significantly over the study period (*p* = .02), with a small effect size (*d =* 0.4). Similarly, mean scores on the HAP indicated engagement in physical activity also increased. At T1, participants were engaging in more strenuous activities than they were at T0 (*p* = .01), and more physical activity overall (*p* = .02), though effect sizes were negligible (*d*s = 0.2), with improvements concentrated in entertainment/social and independent exercise domains.

### Health-related quality of life

Participants demonstrated significant improvement in physical aspects of HRQoL over the study period. Overall physical wellbeing (PCS) improved significantly (*p* = .01), with a small effect size (*d* = 0.30), while there was no significant change in mental aspects of HRQoL (MCS; *p* = .43, *d* = 0.11).

### Communication with healthcare providers

Participants’ self-reported communication with HCPs improved significantly between T0 and T1 (*p* = .01), with a small effect size (*d* = 0.3).

### Fruit and vegetable consumption, alcohol use, and smoking

Fruit and vegetable intake improved significantly over the study period, evidenced by self-reported serves consumed on the day prior to assessment (*p* < .001), with a large effect size (*d* = 1.7). Alcohol consumption decreased significantly (*p* = .01), though effect size was negligible (*d* = 0.1). There was no change in cigarettes per day amongst the four smokers.

### Emotional distress

There were significant reductions in overall emotional distress as assessed by the DASS-21 between T0 and T1 (*p* < .001), with a large effect size (*r* = .7). These improvements were concentrated in the areas of depression (*p* = .03) and stress (*p* = .01), with medium effect sizes for both subscales (*r*s = .3).

### Blood pressure

T0 and T1 BP data was available for 39 participants. Significant improvement was seen in systolic (*p* < .01) and diastolic (*p* = .02) measurements, with medium effect sizes (*d*s = 0.7 and 0.6, respectively). Percentage of participants at target (≤120/80) remained stable (88.5%).

## Discussion

Through a person-centred, theory-based approach to SMS, this exploratory study demonstrates improved behavioural and patient outcomes among people with stages 1–4 CKD. As postulated by SCT, this was associated with improvements in knowledge, emotional distress, and self-efficacy. The person-centred, flexible approach meant not only that a wide range of people (including those with physical disabilities such as quadriplegia, blindness, and limb amputation and those with English as their second language, as well as full-time workers with busy schedules) were able to participate, but also that personally meaningful goals were able to be worked towards in order to achieve overall improvements in self-efficacy and self-management. PCC as standard in SMS would mean support was directed where it was needed, a more efficient use of time and resources. The heart failure (HF) literature provides support for this idea, with studies demonstrating that, when delivered as intended, PCC improves patient outcomes and decreases disease burden [47, 48].

Individuals are experts on their lives, yet people with chronic disease often do not feel that HCPs value their knowledge and insight regarding their condition [49]. In this study, those with lower self-efficacy at baseline chose more intensive intervention schedules, demonstrating awareness of need for support. SMS in CKD has historically been delivered and evaluated from the perspective of HCPs, focusing on what they know to be important and assuming that provision of disease-specific information will lead to improved self-management [8, 50–52], while failing to account for the complexity of chronic disease self-management from the perspective of their patients. Multiple reviews of CKD self-management intervention studies have been published in recent years [8–10, 53], however, they consistently reach similar conclusions: that studies are limited and difficult to review due to large variation in samples, methodologies, and outcomes. In chronic disease, the burden of disease-management is overwhelmingly with the individual, and it is crucial that support processes are in place to set up and maintain effective self-management. In the field of SMS for these diseases, meaningful change for individuals is what is important, and flexibility in intervention and evaluation protocols is going to be necessary in order for this support to reach those who need it most. In contrast to repeated attempts to synthesise overall results of studies in this field, meta-analyses of individual patient data provide techniques which can help to ascertain what works for whom, and under what conditions, rather than continually trying to synthesise disparate studies [54].

After receiving the individualised CKD-SMS, participants displayed overall improvements on several outcomes. Both primary outcomes improved significantly, with small to medium effect sizes. We also found significant improvement in several secondary outcomes. The pattern of findings is consistent with a SCT model of CKD self-management [17, 55, 56], indicating that the intervention led to changes in knowledge and self-efficacy via multiple sources (education, performance accomplishments, vicarious experience, verbal persuasion, and self-appraisal) which led to changes in behaviour and outcomes. Testing of our SCT model was beyond the scope of this study, but it has provided a framework for future research by proposing a model for self-management in CKD which is empirically testable using standardised measures. In addition to elements assessed in this study, several participants desired assistance with sleep (i.e., training in sleep hygiene), and this was provided during intervention sessions and by way of additional resources. This may be an area that is important to include as an outcome in future studies.

Inclusion of goal-setting and awareness of general SMS needs in planning and implementing person-centred intervention is crucial, as it has been identified that outcome measures often do not even match goals of people with chronic diseases [57]. On an individual, goal-focused level, the areas of greatest improvement were for knowledge and overall self-management. That said, all participants demonstrated improvement in one or more outcomes, indicating that person-centred intervention has the potential to improve patient outcomes overall. This is consistent with previous research across various chronic diseases, which has determined that interventions that are tailored to patient activation [58], preferences [47], values and goals [59], and/or individual circumstances [60] and aligned with principles of PCC [61] can lead to improved patient outcomes. Additionally, HF research has indicated that compatibility of prescribed self-management tasks with life goals is unsurprisingly associated with adherence [62].

The findings of this study are consistent with those of previous research which have indicated that self-management interventions can lead to improved outcomes (see [8–10, 53] for reviews and limitations). Improvements in BP provide physiological evidence of participant-reported behaviour change, although, as renal function deteriorates, more pharmacological intervention to control BP is required. Smoking behaviour did not improve, however only four smokers participated, and each indicated an unwillingness quit. Smoking cessation is challenging, requiring both readiness and often intensive, targeted intervention [63]. Despite positive outcomes, kidney function declined during the study, although there are multiple possible explanations for this. First, it must be noted that T1 eGFR was only available for participants who were required to see their nephrologist at least every 3 months (*n* = 46), indicating faster kidney function decline. Second, effect from lifestyle modifications takes significant time – longer than the three-month follow-up in this study [64]. Third, understanding about those who are likely to experience CKD progression is emerging, and interventions to slow decline warrant further research.

This research indicates that delivery of individualised, person-centred, theory-based self-management support has potential to help patients with CKD to achieve clinical targets and better health and quality of life outcomes. While our focus was the development of a person-centred intervention, future implementation research is needed to examine its scalability in clinical practice. Elsewhere we report on participants’ perspectives of the CKD-SMS intervention, which support this approach as highly useful and helpful in managing their CKD [65]. Yet we acknowledge the systems barriers to change that reduce uptake of person-centred innovation in healthcare [66]. There are opportunities for this intervention to be delivered at existing nurse-led CKD clinics (e.g., [67]), or by nurses alongside routine clinical appointments at outpatient clinics. There is also potential for practices such as this to attract additional funding, with the current international focus on PCC in healthcare practice [68, 69]. Self-management support desires of people with CKD identified by our previous work [20, 21] could also be used in healthcare contexts to guide goal-setting and development over time. Those with poorer relationships with their HCPs and greater complexity in terms of treatment regimens and comorbidities are likely to need more support than those who feel supported by their HCPs and/or have less complex health problems. People with CKD have to live and manage their condition in an environment fraught with illness and treatment complexity and inconsistency. Person-centred care provides an opportunity to support patients within their complex healthcare environment, and findings from the HF literature demonstrating that PCC helps alleviate feelings of illness-related complexity and ambiguity indicate that it can be effective in doing so [70]. Recognition that people with chronic disease frequently suffer from multiple comorbidities (increasing complexity and rendering advice focused on one discrete illness unhelpful [71]) is also crucial. Government bodies are starting to recognise the importance of integrated care, and to provide subsidies for a HCP in a patient advocate role who has a holistic understanding of individual patients [72].

This study was limited by the fact that data were collected by the researcher who delivered the intervention, which may have encouraged response bias. Selection bias is also possible due to the study design, and may have favoured patients who were motivated to engage in self-management. We were unable to report data on non-consenting patients, however we would expect the intervention effects to be even greater in a more diverse sample. A further limitation of the study was its pre-post design, which does not allow for comparison to an active control group (e.g., one in which participants receive pure information with no SCT or PCC elements). There is an opportunity for future research to build on this study, using longer term evaluations of programs such as the CKD-SMS to assess maintenance of behaviour change and effects on clinical outcomes and disease progression, and also to investigate effects of an intervention such as this in comparison to an active control group. Other constructs not captured in this study such as social support may also be built into the SCT model proposed here.

## Conclusions

CKD research has generally proceeded in ways that ignore the complexity of self-managing chronic illness – both in the design of interventions and the reporting of study outcomes. In doing so, it has fallen short of developing models that are meaningful to people with CKD, and failed to provide practitioners with the kind of knowledge needed to best support patients. There are several systematic reviews that are inconclusive or do not provide information about how to optimise SMS to meet patient needs. This study is an important step in moving the field forward: demonstrating improved outcomes in early stage CKD by adopting a person-centred approach to SMS. It also supports SCT as a useful framework to guide future interventions. People with CKD have diverse needs and associated complex comorbidity. It is important to consider individual circumstances, needs and goals, as well as current level of activation, when providing SMS to this population.

## Additional files

Additional file 1: CKD-SMS 12-Week Program Procedure. (PDF 60 kb)

Additional file 2: Clinical Examples of CKD-SMS Strategies. (PDF 58 kb)

## Abbreviations

BP: Blood pressure
CKD: Chronic kidney disease
CKD-SMS: The chronic kidney disease self-management support intervention
ESKD: End-stage kidney disease
HF: Heart failure
KRT: Kidney replacement therapy
PCC: Person-centred care
SCT: Social-cognitive theory

## Footnotes

1 Handbook and handouts available from lead author upon request.
