# Supplementary material for: Meeting patients where they are: improving outcomes in early chronic kidney disease with tailored self-management support (the CKD-SMS study)
Source: BMC Nephrol. 2018 Oct 20;19:279. doi: 10.1186/s12882-018-1075-2 (PMC6195997; doi:10.1186/s12882-018-1075-2)
Supplement: Supplementary file 1 — CKD-SMS 12-Week Program Procedure. (PDF 60 kb) [file 12882_2018_1075_MOESM1_ESM.pdf]

Additional File 1: CKD-SMS 12-Week Program Procedure.

Week	Main Purposes	Procedure
1 – Face-to-Face Session	• Goal-setting<br>• Education<br>• Planning	1. Spend time building rapport & getting to know participant.<br>2. Ask participant if they have pre-existing self-management goals.<br>3. Use companion handbook and handouts as a guide to lead conversation. Allow participant to guide areas of focus, which may include:<br>a. CKD & kidney knowledge<br>b. Medication management<br>c. Healthy eating<br>d. Routine & planning<br>e. Communication with HCPs<br>f. Physical activity<br>g. Mental health/emotional distress<br>h. Symptom-management<br>i. Maintaining social & occupational roles<br>j. Engaging social support<br>4. Develop & refine meaningful & achievable goals.<br>5. Create plan & problem-solve any perceived potential barriers.<br>6. Training in any relevant self-monitoring.<br>7. Agreement upon frequency of phone sessions.
2-11 – Telephone Support Sessions	• Reinforcement of successful self-management behaviour<br>• Problem-solving and planning	1. Spend time re-establishing rapport and discussing how the participant has been during the period between sessions.<br>2. Enquire as to whether there are any particular issues the participant would like to discuss today. If yes, spend time on these.<br>3. Discuss tasks agreed upon during previous session – what worked/did not work and why.<br>4. Reinforce successful performance of effective self-management behaviours & problem-solve regarding any barriers which may have emerged.<br>5. Set plan and goals for the coming period & problem-solve any perceived potential barriers.<br>6. Teach participant to access any additional useful resources or, if not possible, email to participant.<br>7. Agree upon timing of next follow-up.
12 – Face-to-Face Session	• Reinforcement of successful self-management behaviour<br>• Problem-solving and future-planning	1. Spend time re-establishing rapport and discussing how the participant has been during the period between sessions.<br>2. Enquire as to whether there are any particular issues the participant would like to discuss today, and if there are any areas that they feel have not been covered by the CKD-SMS so far. If yes, spend time on these.<br>3. Discuss tasks agreed upon during previous session – what worked/did not work and why.<br>4. Reinforce successful performance of effective self-management behaviours and progress made during the program.<br>5. Future planning – discuss and problem-solve regarding maintenance of changes now that the program is concluding.<br>6. Teach participant to access any additional useful resources or, if not possible, provide any final resources.
