# Supplementary material for: Meeting patients where they are: improving outcomes in early chronic kidney disease with tailored self-management support (the CKD-SMS study)
Source: BMC Nephrol. 2018 Oct 20;19:279. doi: 10.1186/s12882-018-1075-2 (PMC6195997; doi:10.1186/s12882-018-1075-2)
Supplement: Supplementary file 2 — Clinical Examples of CKD-SMS Strategies. (PDF 58 kb) [file 12882_2018_1075_MOESM2_ESM.pdf]

**Additional File 2:** Clinical Examples of CKD-SMS Strategies.

| Strategy | Purpose | Example |
|---|---|---|
| SCT strategies (performance accomplishment reinforcement, vicarious experience, verbal persuasion) | • To increase self-efficacy to engage in effective self-management behaviours | A morbidly obese participant was aware that they were eating poorly, but felt unmotivated to change due to previous experiences with yo-yo dieting and weight loss and regain, and their perception that a person must eat "perfectly" and very little in order to gain any benefit. This participant was educated regarding benefits of healthy eating beyond possible effects on weight (in particular, they were also struggling with poorly managed diabetes and cardiovascular problems), and encouraged to discuss how they had felt when they had eaten a diet including fruits and vegetables in the past. They were able to identify that they felt better, and that their blood sugar levels were more easily managed and their cholesterol lower. This person was asked whether they were able to identify changes which were likely to be achievable and not too burdensome. Barriers (including lack of time and interest in cooking) were identified, and strategies for overcoming these (such as individually packaged single serves of microwave steamed vegetables) were identified and implemented. Over the course of the program, this participant was able to gradually increase their vegetable intake. |
| Cognitive-behavioural strategies | • Depression<br>• Anxiety<br>• Stress<br>• Insomnia/sleeping difficulty | One participant was suffering from symptoms of generalised anxiety, primarily excessive worry and difficulty sleeping. In addition to training in mindfulness exercises (discussed further below), the participant was taught strategies including problem-solving instead of worrying, challenging unhelpful thoughts, and setting aside dedicated "worry time" (and postponing worries until that time). Education regarding insomnia was also provided, along with training in sleep hygiene and sleep monitoring. By the end of the CKD-SMS, the participant reported feeling that they had the necessary tools to make changes to their worrying and poor sleep habits. |
| Mindfulness exercises | • Depression<br>• Anxiety<br>• Stress<br>• Insomnia/sleeping difficulty | A participant who was living in a nursing home was experiencing feelings of stress and difficulty winding down to sleep. They were severely visually impaired, and therefore unable to use written materials. During sessions, this participant was talked through multiple different mindfulness exercises several times, and they identified their favourites which best helped them wind down. One example is the "Leaves on a Stream" exercise, where a person imagines lying by a stream on which leaves are floating past. The person clears their mind and, when thoughts drift into their consciousness, they visualise placing them on the leaf, and watching them drift away. At the conclusion of the program, this participant was given an audio CD with several of their favourite mindfulness exercises recorded upon it, so that they may continue to do these on their own. |
| Motivational interviewing | • Participant ambivalence regarding behavioural changes | Someone who was working full time expressed awareness that they did no physical activity, but hopelessness regarding whether it would have any positive impact and feelings that they had no time for it anyway. Forms of physical activity that the participant had enjoyed in the past were discussed, as were the positive impacts they experienced from it during those times. Pros and cons of engaging in a small amount of physical activity (playing with and/or walking the dog after work) were discussed with the participant, and they realised that doing so would most likely have a positive impact upon his life on multiple levels. As the program went on, this participant identified that they were feeling fitter and healthier, had lost a small amount of weight, and were enjoying spending time with their dog. |
